# Design and Biological Evaluation of hBest1-Containing Bilayer Nanostructures

**DOI:** 10.3390/molecules30142948

**Published:** 2025-07-12

**Authors:** Pavel Bakardzhiev, Teodora Koleva, Kirilka Mladenova, Pavel Videv, Veselina Moskova-Doumanova, Aleksander Forys, Sławomira Pusz, Tonya Andreeva, Svetla Petrova, Stanislav Rangelov, Jordan Doumanov

**Affiliations:** 1 Institute of Polymers, Bulgarian Academy of Sciences, Akad. G. Bonchev Str. 103A, 1113 Sofia, Bulgaria; 2 Faculty of Biology, Sofia University “St. Kliment Ohridski”, Dragan Tsankov Blvd., 1164 Sofia, Bulgaria; teodorakoleva21@gmail.com (T.K.); k_mladenova@biofac.uni-sofia.bg (K.M.); moskova@biofac.uni-sofia.bg (V.M.-D.);; 3 Centre of Polymer and Carbon Materials, Polish Academy of Sciences, ul. M. Curie-Skłodowskiej 34, 41-819 Zabrze, Poland; aforys@cmpw-pan.pl (A.F.);; 4 Faculty Life Sciences, Reutlingen University, Alteburgstraße 150, 72762 Reutlingen, Germany; tonya.andreeva@reutlingen-university.de; 5 Institute of Biophysics and Biomedical Engineering, Bulgarian Academy of Sciences, “Acad. G. Bonchev” Str. 21, 1113 Sofia, Bulgaria

**Keywords:** hBest1, bilayer nanostructures, MDCK II cells

## Abstract

Bestrophinopathies are a group of inherited retinal diseases caused by mutations in the *BEST1* gene. The protein encoded by this gene, bestorphin-1 (hBest1), is a calcium-dependent transmembrane channel localized on the basolateral membrane of retinal pigment epithelial (RPE) cells. We have already demonstrated the surface behavior and organization of recombinant hBest1 and its interactions with membrane lipids such as 1-palmitoyl-2-oleoyl-sn-glycero-3-phosphocholine (POPC), sphingomyelin (SM) and cholesterol (Chol) in models of biological membranes, which affect the hBest1 structure–function relationship. The main aim of our current investigation is to integrate pure hBest1 protein into lipid bilayer nanostructures. We synthesized and characterized various hBest1-containing nanostructures based on 1,2-Dipalmitoylphosphatidylcholine (DPPC), SM, glycerol monooleate (GMO) and Chol in different ratios and determined their cytotoxicity and incorporation into cell membranes and/or cells by immunofluorescence staining. Our results show that these newly designed nanoparticles are not cytotoxic and that their incorporation into MDCK II cell membranes (used as a model system) may provide a mechanism that could be applied to RPE cells expressing mutated hBest1 in order to restore their ion transport functions, affected by mutated and malfunctioning hBest1 molecules.

## 1. Introduction

**Scheme 1:**
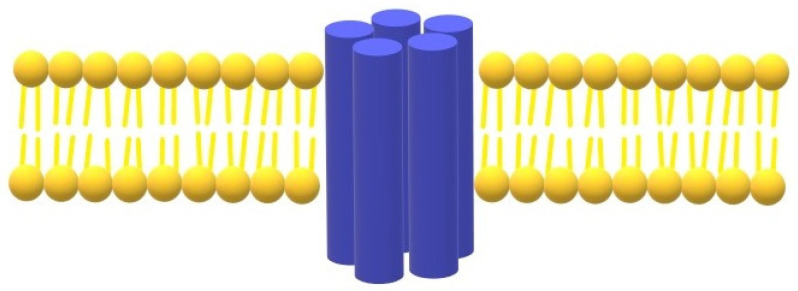
Illustration of the pentameric structure of hBest1 (in blue). Lipids are in yellow.

Bestrophinopathies are a group of inherited retinal diseases caused by mutations in the *BEST1* gene [1,2] which include classical Best vitelliform macular dystrophy (BVMD) [3,4], adult-onset vitelliform macular dystrophy (AVMD), autosomal dominant vitreoretinochoroidopathy (ADVIRC), autosomal recessive bestrophinopathy (ARB) [5] and retinitis pigmentosa (RP) [6,7]. The protein encoded by this gene, bestorphin-1 (hBest1), is a calcium-dependent transmembrane chloride channel localized on the basolateral membrane of retinal pigment epithelial (RPE) cells [2,6,8,9,10,11] that can also transport glutamate and γ-aminobutyric acid across the plasma membrane of cells [12,13,14,15,16]. Owji et al. presented a pentameric structure of hBest1 [17], previously also shown for KpBest and cBest1 [18,19]. A schematic illustration of the pentameric structure of hBest1 is presented in Scheme 1.

We have already demonstrated the surface behavior and localization of recombinant hBest1 in the cell membranes (in lipid rafts and non-rafts) of eucaryotic MDCKII cells, and its miscibility and phase separation with different membrane lipids, such as 1-palmitoyl-2-oleoyl-sn-glycero-3-phosphocholine (POPC), sphingomyelin (SM) and cholesterol (Chol), in designed biological membrane models that reshape its self-organization and conformational states due to multiple non-covalent protein–lipid interactions crucial for the structure–function relationship of hBest1 [20,21,22,23,24]. This knowledge led us to design lipid vesicles containing the protein, such as liposomes, and to exploit their ability to integrate hBest1 protein into cell plasma membranes, thereby restoring impaired membrane function due to mutant and non-functional forms of bestrophin-1.

Liposomes are self-assembled vesicular structures composed of one or more lipid bilayers enclosing an aqueous cavity [25,26]. Driven by thermodynamic forces, they are formed spontaneously when phospholipid molecules are dispersed in an aqueous solution. Liposomes are mostly spherical or nearly spherical in shape and less commonly non-spherical (cuboidal or hexagonal) [25,27,28,29]. They are classified into several subgroups. According to the number of bilayers, they are classified as ULVs (unilamellar vesicles) and MLVs (multilamellar vesicles) [25,30,31]. According to their size, unilamellar vesicles are divided into SUVs (small unilamellar vesicles), LUVs (large unilamellar vesicles) and GUVs (giant unilamellar vesicles) [32,33,34,35]. Due to their biophysical properties such as charge and small size, liposomes are widely used in drug delivery. Their amphiphilic character also allows the loading of hydrophilic molecules into the liposome cavity or the integration of hydrophobic molecules into the phospholipid bilayer. Liposomal nanocarriers provide a barrier function for the drug loaded into the cavity, thus preventing any side biological or chemical reactions with the surrounding environment. Another advantage that makes liposomes reliable drug carriers is their low cytotoxicity due to the fact that they are composed of lipids found naturally in cell membranes [32,36,37,38,39].

The type of liposomes’ lipid constituents determines their characteristics such as fluidity, charge and fusogenic potential [36]. The design and construction of nanoparticles using biological lipids can provide a favorable environment for integrated membrane proteins (dopamine D2L receptor or bacteriorhodopsin), similar to that in the natural cell membrane [40,41], allowing them to retain their native conformations [27]. The ability of liposomes to fuse with cell membranes is important for delivering any hydrophilic, hydrophobic or amphiphilic molecule. For hydrophilic drugs, this ability allows lysosomal escape and direct release of the cargo in the cytosol. The use of fusogenic liposomes or those with a cubic phase tendency favoring fusion with the cell membrane opens up possibilities for delivering functionally active membrane proteins to cells whose membranes contain mutant channel forms associated with pathological conditions (channelopathies) [42,43]. These nanocarriers are a prerequisite for the development of modern therapies for a group of diseases associated with the dysfunction of ion channels localized in cell membranes, such as bestrophinopathies caused by mutant forms of hBest1 protein.

We previously generated MDCKII-hBest1 cells that stably express hBest1 and optimized a method for the purification and isolation of the protein [20]. Here, we report the formation of the first lipid uni- or multilamellar nanostructures containing hBest1 protein and their ability to deliver hBest1 to the cell membrane of target MDCKII cells. Physicochemical characterization and biological evaluation of the resulting nanostructures by cryogenic transmission electron microscopy (cryo-TEM), MTT analysis and immunofluorescence staining are performed and discussed.

## 2. Results

### 2.1. Design and Self-Association of Lipid Nanostructures Containing hBest1 Protein

**Scheme 2:**
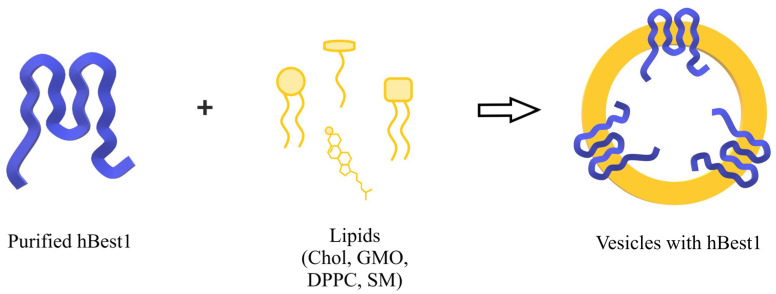
Schematic presentation of the formation of vesicles containing hBest1.

We previously reported cuboidal vesicular structures containing DPPC, Chol and polyglycidol-derivatized lipid mimetics and hypothesized the gradual transition to bilayer membranes of zero curvature and the fluidization of the membranes [28]. Here, we changed the content of the structures, which, instead of polymer conjugates, now contain pure hBest1 protein, isolated and purified from MDCKII-hBest1 cells as we have already shown [20]. The vesicles were prepared by the direct hydration of lyophilized mixtures of DPPC, Chol and SM or GMO (glycerol monooleate) with a pure buffer solution or with a buffer solution containing hBest1, as schematically presented in Scheme 2.

**Figure 1:**
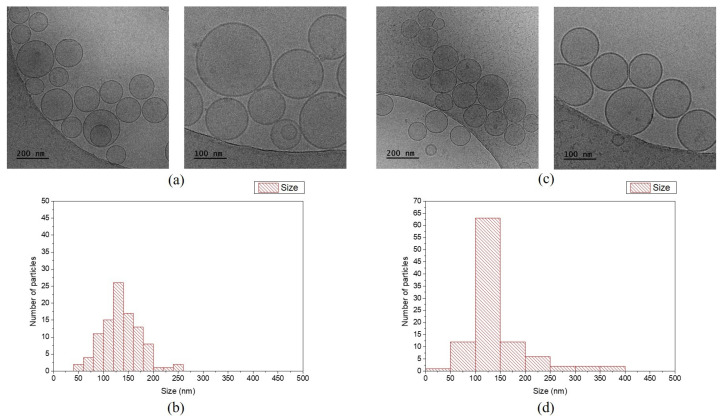
Cryo-TEM images of DPPC/SM/Chol vesicles with sizes 50–250 nm, some of them multivesicular (**a**): average size (measured for 100 objects) of 135 nm, with size distribution in histogram below (**b**); and DPPC/Chol/GMO vesicles with sizes 40–380 nm, some of them multivesicular (**c**): average size (measured for 100 objects) of 145 nm, with size distribution in histogram below (**d**). Thickness of all membranes: ~5 nm. All vesicles contain 0.15 mol % of hBest1.

**Figure 2:**
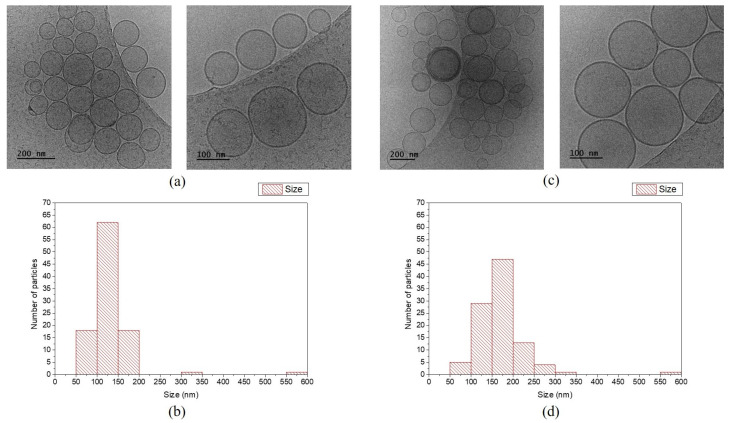
Cryo-TEM images of SM/Chol vesicles with sizes 50–200 nm, some of them multivesicular (**a**), with small number of particles with sizes over 200 nm (300–600 nm) and average size (measured for 100 objects) of 130 nm, with size distribution in histogram below (**b**); and DPPC/Chol vesicles with sizes 80–315 nm, some of them multivesicular or multilamellar (**c**), with small number of particles with sizes over 200 nm (300–600 nm) and average size (measured for 100 objects) of 171 nm, with size distribution in histogram below (**d**). Thickness of all membranes: ~5 nm. All vesicles contain 0.15 mol % of hBest1.

Four types of vesicles containing 0.15 mol% hBest1 were prepared: (1) DPPC/Chol/SM with a molar ratio of 1/1/1; (2) DPPC/Chol/GMO with a molar ratio of 1/1/1; (3) SM/Chol with a molar ratio of 2/1; and (4) DPPC/Chol with a molar ratio of 2/1. Cryo-TEM was employed to visualize vesicle morphology and size. The dominant nanostructures in all ratios were spherical and unilamellar, but some of them were multilayer vesicles. The average size of the four-component vesicles is very narrow (between 135 and 145 nm), while that of the three-component ones is between 130 and 171 nm. In all structures, the membrane thickness is ~5 nm (Figure 1 and Figure 2), which corresponds well to the membrane thickness in the gel state for DPPC of about 4.6 nm [44].

### 2.2. Biological Evaluation of hBest1 Lipid Nanostructures

The biological significance of hBest1 lipid nanostructures in terms of their effect on cellular metabolic activity and toxicity, as well as the cellular uptake of the hBest1 protein, was evaluated.

#### 2.2.1. Metabolic Activity Assessment of MDCK II Cells Treated with hBest1 Lipid Nanostructures

**Figure 3:**
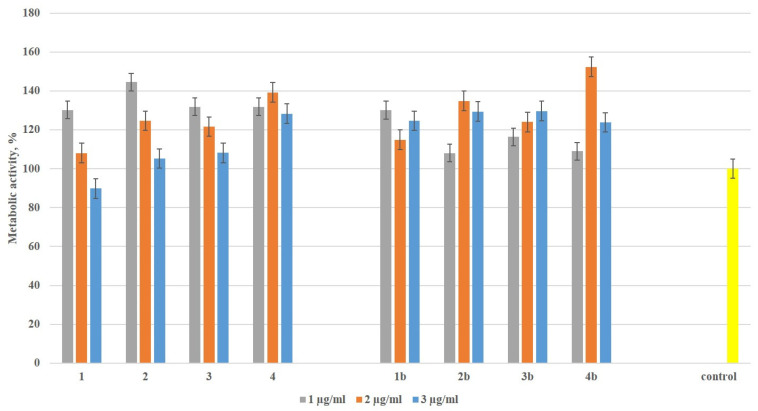
Metabolic activity defined via the MTT test after 24 h of incubation of MDCK II cells treated with different concentrations of (1) DPPC/Chol/SM, (2) DPPC/Chol/GMO, (3) SM/Chol and (4) DPPC/Chol vesicles as well as 1b, 2b, 3b and 4b vesicles containing, in addition to the same ratio of lipids, the protein hBest1. The data are presented as percentages of control cells as the mean ± SE; *n* = 3.

The MTT test was performed to evaluate MDCK II cells’ metabolic activity after 24 h of incubation with various types of lipid nanovesicles at different concentrations (1, 2 and 3 μg/mL). First, the non-toxicity of the hBest1-free lipid vesicle to MDCK II cells was assessed (Figure 3). The metabolic activity of MDCK II cells treated with increasing concentrations of vesicle types 1, 2 and 3 demonstrated concentration-dependent inhibition, not observed after cell treatment with vesicle type 4 (Figure 3). Only vesicle type 1 caused low cytotoxic effects on cells at a concentration of 3 μg/mL, inducing about 20% inhibition of metabolic activity. All other lipid vesicles showed an increase in metabolic activity from 10% to 50%. Second, to assess the potential of designed nanostructures as safe carriers of hBest1 protein, we determined the metabolic activity of MDCK II cells treated with increasing concentrations of novel vesicles containing hBest1, designated as 1b, 2b, 3b and 4b hBest1. All particles caused a biologically significant change associated with an increase in metabolic activity from 10% to about 50% (Figure 3), most probably due to the integration of hBest1 and the fusion of vesicles with plasma membranes. These results indicate that hBest1 and hBest1-free vesicles displayed no cytotoxic effect at all used concentrations.

#### 2.2.2. Immunofluorescence Staining of hBest1 Protein in MDCK II Cells Treated with hBest1 Vesicles

**Figure 4:**
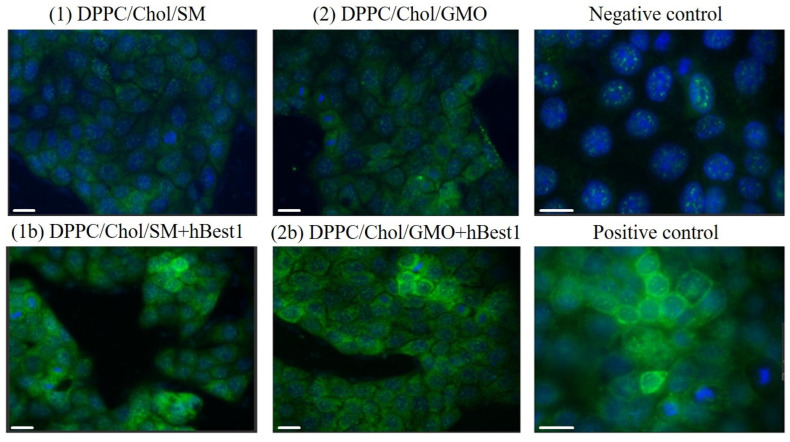
Immunofluorescence staining of hBest1 (green) in MDCK II cells treated for 2h with (1b) DPPC/Chol/SM and (2b) DPPC/Chol/GMO containing hBest1 protein. Nuclei are in blue; scale bar = 15 μm. The concentration of vesicles used in cell treatment was 1.5 µg/mL relative to hBest1. Negative control—MDCK II cells. Positive control—MDCK II-hBest1 cells.

To examine the cellular uptake of the hBest1 protein, MDCK II cells were incubated for 2 h with vesicles 1b, 2b, 3b and 4b, and immunofluorescence was applied to detect the hBest1 signal. In the cells incubated with 1b and 2b vesicles, a green hBest1 signal was detected, whereas in the cells treated with 3b and 4b vesicles, the signal was not detectable or was too weak to be evaluated, similar to the negative control of MDCK II cells (Figure 4). The morphology, shape and size of the cells did not change after incubation with the nanostructures and displayed normal characteristic features of control cells, which is consistent with previous results (Figure 3 and Figure 4). The hBest1 fluorescence signal observed in the cells incubated with 1b and 2b vesicles remained most intense in the cell membrane area, similar to MDCK II–hBest1 cells (positive control), where hBest1 is localized at the cell periphery and along the plasma membrane. This is proof that the hBest1 protein, or at least a large portion of it, is directly integrated into the cell membrane upon membrane fusion of hBest1-containing nanostructures.

## 3. Discussion

Direct delivery of the transmembrane hBest1 protein to the plasma membranes of cells is a promising approach for the treatment of bestrophinopathies. This approach involves the use of cubosomes and/or liposomes with encapsulated hBest1 protein in the lipid phase of the vesicles and their fusion with the cell membrane. Similar experiments in incorporating a transmembrane protein into a lipid cubic phase have been carried out with bacteriorhodopsin and D2L dopamine receptor [40,41,45]. Ramadan et al., 2020, have already reported the ability of liposomes to deliver glycine receptor (GlyR) to the cell membrane of the target cell [42,43].

Here, for the first time, we report the construction of lipid vesicles containing hBest1. Vesicles contain lipids that are natural for biological membranes, such as SM, Chol and DPPC, as well as the nonlamellar lipid GMO, which is not found in cell membranes. Cryo-TEM images of DPPC/Chol/SM/hBest1 and DPPC/Chol/GMO/hBest1 reveal that they have an average size of 135 nm and 145 nm, respectively; i.e., there is a difference in average size of about 10 nm (Figure 1). This may be due to the balance between the condensing effect of cholesterol (strongly pronounced for SM, hBest1 and DPPC) and the disruption of membrane order caused by the nonlamellar GMO [23,46,47,48]. For the ternary vesicles SM/Chol/hBest1 and DPPC/Chol/hBbest1, the average size is 130 nm and 171 nm, respectively, with the size difference increasing significantly and being about 40 nm (Figure 2).

The negative Δ*G* values of the SM/Chol/hBest1 monolayers indicate that the mixing of hBest1 and lipid molecules is a spontaneous and thermodynamically favorable process [24]. We also showed that the condensing effect of Chol is most prominent in the ternary SM/Chol/hBest1 monolayers [23]. Both results indicate the very good packing and arrangement of lipid and protein molecules in the membranes, which also result in the significantly smaller size of the SM/Chol/hBest1 vesicles compared to DPPC/Chol/hBest1.

Next, it was important to evaluate the in vitro metabolic activity of MDCK II cells treated with increasing concentrations of hBest1 vesicles, comparing it with the metabolic activity of cells treated with hBest1-free lipid vesicles (Figure 3). The overall increase in the metabolic activity of the treated cells determines the lack of cytotoxicity of all tested vesicles, indicating their biocompatibility. The observed metabolic fluctuations depend on numerous factors such as cell types, cell membrane composition, the cell metabolic response to nanostructures and their concentrations, plasma membrane penetration disturbances, diffusion limits, basic fusion steps upon association, etc. The long-term biocompatibility or potential side effects of obtained hBest1 lipid vesicles will be investigated and analyzed in depth, but our first achievements are promising.

In previous studies, we used different cell types, such as HepG2, A549 and MDCK II, for biological evaluation of different nanoparticles [49]. The MDCK II cell line originates from non-cancerous tissue (kidney epithelium) and is one of the most widely used models for studying cell polarization and lipid rafts. MDCK II cells are similar to retinal pigment epithelial cells, although these cells do not naturally express hBest1 protein [11].

Previously, we established a cell line of MDCK II cells that stably expressed hBest1 [50], and now, we have purified the protein [20] from these cells in order to incorporate it into the vesicles that we are currently studying. Due to the lack of endogenous expression of hBest1, the detection of the hBest1 signal by immunofluorescence staining was easy to perform because the antibody only bound to the protein in cells that was transported by vesicles. A typical immunofluorescence signal for hBest1, similar to the positive control from MDCK II-hBest1 cells, was observed in MDCK II cells incubated for 2 h with DPPC/Chol/SM/hBest1 and DPPC/Chol/GMO/hBest1. The majority of the hBest1 signal outlined the cell boundaries, demonstrating its membrane localization (Figure 4). The incorporation of a fusogenic lipid GMO [43] did not lead to better results in DPPC/Chol/GMO/hBest1 vesicles. Since larger liposomes are generally considered to be the reason for their higher loading capacity [51], it could be assumed that liposomes with larger diameters could exhibit an increased capacity to incorporate hBest1 protein, but our results did not show this trend. In stably transfected MDCK-hBest1 cells, hBest1 was distributed between liquid-ordered (L_o_, 35%) and liquid-disordered (L_d_, 65%) fractions [22]; therefore, one of the possible reasons for obtaining the hBest1 signal from cells incubated with the three-component lipid (DPPC/Chol/SM and DPPC/Chol/GMO) vesicles is that they simulate both liquid-ordered and liquid-disordered regions in the cell membranes, whereas the two-component ones (SM/Chol and DPPC/Chol) simulate such regions separately.

Whether the structure and functionality of hBest1 are preserved in vesicles and in cell membranes, what the mechanism of fusion and protein delivery in eukaryotic cells is, what the efficiency of fusion is, and whether the integrated protein can restore membrane functions in cells affected by mutated and malfunctioning hBest1 molecules are questions that this study has raised and that will be answered in the future.

## 4. Materials and Methods

All reagents and chemicals were purchased from Sigma-Aldrich (St. Louis, MO, USA) unless otherwise indicated.

### 4.1. Preparation of Nanostructures Containing hBest1 Protein

DPPC, Chol, SM, GMO and methanol were purchased from Sigma Apdrich. Methanol solutions of DPPC, Chol, SM and GMO in desired molar ratios and a total lipid concentration of 3 mM were mixed and placed into glass tubes. The mixed solutions were flash-frozen in liquid nitrogen and lyophilized. The resulting lyophilisates were directly hydrated with PBS or PBS containing hBest1.

### 4.2. Cryogenic Transmission Electron Microscopy (Cryo-TEM) Measurements

Cryogenic transmission electron microscopy (cryo-TEM) images were obtained using a Tecnai F20 X TWIN microscope (FEI Company, Hillsboro, OR, USA) equipped with a field emission gun, operating at an acceleration voltage of 200 kV. Images were recorded on the Gatan Rio 16 CMOS 4k camera (Gatan Inc., Pleasanton, CA, USA) and processed with Gatan Microscopy Suite 3.31.2360.0 (GMS) software (Gatan Inc., Pleasanton, CA, USA). Specimen preparation was conducted by the vitrification of the aqueous solutions on grids with holey carbon film (Quantifoil R 2/2; Quantifoil Micro Tools GmbH, Großlöbichau, Germany). Prior to use, the grids were activated for 15 s in oxygen plasma using a Femto plasma cleaner (Diener Electronic, Ebhausen, Germany). Cryo-samples were prepared by applying a droplet (3 μL) of the suspension to the grid, blotting it with filter paper and immediately freezing it in liquid ethane using the fully automated blotting device Vitrobot Mark IV (Thermo Fisher Scientific, Waltham, MA, USA). After preparation, the vitrified specimens were kept under liquid nitrogen until they were inserted into a cryo-TEM-holder Gatan 626 (Gatan Inc., Pleasanton, CA, USA) and analyzed in the TEM at −178 °C.

### 4.3. Cell Cultures

MDCK cells (ATCC, Teddington, UK), strain II, were grown in DMEM with 10% FCS, streptomycin (100 mg/L) and penicillin (60 mg/L) at 37 °C and 5% CO_2_ [50]. Stably transfected MDCK II-hBest1cells were maintained with 500 μg/mL G418 salt [20,50].

### 4.4. hBest1 Purification from MDCK II-hBest1 Cells

The hBest1 stably transfected MDCK II cells were lysed and hBest1 purification was performed as indicated in [20]. The concentration of pure hBest1 protein was determined by the method of Smith et al. [52].

### 4.5. MTT Assay for Metabolic Activity

The metabolic activity of MDCK II cells was assessed using the MTT assay. Cells with an initial concentration of 5 × 10^4^ cells per well were grown for 24 h in 96-well plates and were incubated for 24 h with (1) DPPC/Chol/SM, (2) DPPC/Chol/GMO, (3) SM/Chol or (4) DPPC/Chol vesicles as well as with vesicles containing 0.15 mol% hBest1. The concentrations of the nanostructures used for cell incubation were determined according to the concentration of the hBest1 protein (1, 2 and 3 µg/mL). Cells were treated with particles diluted in serum-free medium. The MTT assay was performed at the 24th hour of vesicle treatment. The analysis was conducted in triplicate.

### 4.6. Immunofluorescence Staining of hBest1 Protein

MDCK II and MDCK II-hBest1 cells with an initial concentration of 5 × 10^4^ cells per well were grown for 24 h in 24-well plates. Standard MDCK II cells (without hBest1) were incubated for 2 h with (1) DPPC/Chol/SM, (2) DPPC/Chol/GMO, (3) SM/Chol or (4) DPPC/Chol vesicles as well as with vesicles containing 0.15 mol% hBest1. The concentration of nanostructures used in incubation with the cells was 1.5 µg/mL, corresponding to hBest1 protein. For the immunofluorescence staining of hBest1, the cells were fixed, blocked and incubated with mouse anti-Best1 (E6-6) (Novus Biologicals Inc., Littleton, France)) for 1 h at room temperature, and bound antibody was detected using secondary goat anti-mouse Alexa Fluor 488 antibody (Abcam, Cambridge, UK) for 1 h at room temperature as described in [11,22]. Nuclei were labeled with DAPI (4′,6-diamidino-2-phenylindole). hBest1 fluorescence was visualized using an GE Healthcare DeltaVisionTM Ultra (Chicago, IL, USA) at 40× magnification.

## 5. Conclusions

The newly designed nanostructures containing hBest1 are non-toxic to MDCK II cells and are capable of transporting and integrating the protein into the plasma membrane of cells. This translational approach forms the core of our research, with future steps aiming to develop functional hBest1 delivery platforms, representing a promising therapeutic strategy for bestrophinopathies.

## Data Availability

Data are contained within the article.

## Abbreviations

The following abbreviations are used in this manuscript:

MDCK	Madin–Darby canine kidney
Chol	Cholesterol
SM	Sphingomyelin
POPC	1-Palmitoyl-2-oleoyl-sn-glycero-3-phosphocholine
